# Association of Maternity Leave Characteristics and Postpartum Depressive Symptoms among Women in New York

**DOI:** 10.1007/s10995-024-03997-1

**Published:** 2024-10-05

**Authors:** Hannah K. Hecht, Angela-Maithy N. Nguyen, Kim G. Harley

**Affiliations:** 1 School of Public Health, University of California, Berkeley, 2121 Berkeley Way, Berkeley, CA 94720-7360 USA; 2 Wallace Center for Maternal, Child, and Adolescent Health, School of Public Health, University of California, Berkeley, 2121 Berkeley Way, Berkeley, CA 94720-7360 USA

**Keywords:** Postpartum depression, Maternal mental health, Maternity leave, Paid parental leave

## Abstract

**Introduction:**

The United States is the only high-income country without a comprehensive national maternity leave policy guaranteeing paid, job-projected leave. The current study examined associations between maternity leave characteristics (duration of leave, payment status of leave) and postpartum depressive symptoms.

**Methods:**

This study used a sample of 3,515 postpartum women from the New York City and New York State Pregnancy Risk Assessment Monitoring System (PRAMS) from 2016 to 2019. We used logistic regression to examine the association of leave duration and payment status with self-reported postpartum depressive symptoms between 2 and 6 months postpartum.

**Results:**

Compared to having at least some paid leave, having unpaid leave was associated with an increased odds of postpartum depressive symptoms, adjusting for leave duration and selected covariates (adjusted odds ratio [aOR] = 1.41, 95% confidence interval [CI]: 1.04–1.93). There was no significant difference in postpartum depressive symptoms between those with partially and those with fully paid leave. In contrast to prior literature, leave duration was not significantly associated with postpartum depressive symptoms (aOR = 0.99, 95% CI: 0.97–1.02 for each additional week of leave).

**Discussion:**

This study suggests that unpaid leave is associated with increased risk of postpartum depression, which can have long-term health effects for both mothers and children. Future studies can help to identify which communities could most benefit from paid leave and help to inform paid leave policies.

**Supplementary Information:**

The online version contains supplementary material available at 10.1007/s10995-024-03997-1.

## Significance

What is already known on this subject?

What this study adds?

Postpartum depression can have long-term consequences for maternal and child health. Longer maternity leave duration and paid maternity leave have been linked with better maternal mental health, though more recent studies in the U.S. context are needed.

This study is one of the few investigations of postpartum mental health to examine leave duration and paid leave individually and in combination. We found that mothers with unpaid leave were 40% more likely to experience depression than those with paid leave and that payment status was more important than leave duration.

**Supplementary Information:**

The online version contains supplementary material available at 10.1007/s10995-024-03997-1.

## Introduction

The United States (U.S.) is the only high-income country without a comprehensive national parental leave policy that guarantees mothers or birthing parents paid, job-protected leave following the birth of a child (UCLA World, 2022). About 56% of the U.S. workforce is eligible for 12 weeks of unpaid, job-protected leave through the Family Medical Leave Act (FMLA) of 1993 (Dagher et al., 2014; KFF, 2021). The recent Federal Employee Paid Leave Act (FEPLA) provides 12 weeks of paid leave for certain federal workers. Given that FMLA is unpaid and FEPLA has strict eligibility criteria, access to paid leave remains limited for new mothers. Only 9 states and DC have paid family leave policies (KFF, 2021). In 2021, only 23% of the U.S. workforce had access to paid family leave, and 40% had short-term disability (KFF, 2021). Moreover, there are demonstrated disparities in access, as having more financial resources is linked with greater access to paid leave and flexibility to take longer leave, even if it is unpaid (Hawkins, 2020; Perry-Jenkins et al., 2017; Slopen, 2020). Ultimately, the lack of a universal paid family leave policy in the U.S. is a public health issue, given the evidence that maternity leave is associated with improvements in maternal and child physical and mental health outcomes during postpartum year and beyond (Andres et al., 2016; Van Niel et al., 2020).

Life course theory posits that early parenthood is a “critical period” for health (Lu & Halfon, 2003). As such, not having temporary leave from work to recover physically and psychologically from giving birth and to bond with one’s infant can exacerbate the stress that accompanies being a parent, leading to poorer mental health (Lee et al., 2020; Saxbe et al., 2018). Paid leave may further protect parental mental health by mitigating the financial stress of taking unpaid leave (Irish et al., 2021).

About 13–19% of women experience postpartum depression (PPD), which includes symptoms such as overwhelm, irritability, and suicidality (O’Hara & McCabe, 2013; Stewart & Vigod, 2016). PPD is associated with subsequent depressive episodes in mothers and can have a negative impact on infant and child development (Slomian et al., 2019). Given these long-term consequences, preventing PPD through programs and policies, such as the expansion of paid maternity leave, is crucial.

Longer maternity leave duration, often operationalized as at least 12 weeks (Chatterji & Markowitz, 2012; Mandal, 2018), is linked with better maternal mental health outcomes (Andres et al., 2016). In a nationally representative cross-sectional study of U.S. women who had worked during pregnancy, each additional week of leave up to 12 weeks postpartum was associated with a lower odds of having postpartum depressive symptoms, while no additional benefit on PPD was seen for additional weeks of leave beyond 12 weeks (Kornfeind & Sipsma, 2018).

Fewer studies have examined the impact of paid versus unpaid leave on postpartum mental health (Aitken et al., 2015; Van Niel et al., 2020). One study found that compared to those without any paid leave, those receiving at least some paid leave had better mental health outcomes, even if they had leave of less than 12 weeks (Mandal, 2018). Meanwhile, in a cross-sectional study of U.S. mothers, taking fully or partially paid leave was associated with better stress management (Jou et al., 2018).

There is a need for further studies of the association between leave characteristics and depression among more diverse populations, especially given demonstrated disparities in access to leave by income and race (Hawkins, 2020; Petts, 2018). Additionally, many existing studies of leave duration do not distinguish between paid and unpaid leave; others rely on data from several decades ago and may not be generalizable to states where leave policies have since evolved. Moreover, much of the research on paid leave examines non-U.S. countries, which tend to have more robust systems to support families that may impact findings (Lee et al., 2020).

In 2016, the state of New York passed a paid family leave (PFL) law providing 12 weeks of job-protected paid time off for employees with a qualifying life event, including the birth of a child (“New York Paid Family Leave,” n.d.). This policy was phased in over four years, beginning in 2018, and applies to most private sector employers (“Eligibility,” n.d.). We examined the associations of duration and payment status of maternity leave with postpartum depressive symptoms among women who gave birth in New York from 2016 to 2019, when the state was undergoing a PFL policy change.

## Methods

### Study Design

The Pregnancy Risk Assessment Monitoring System (PRAMS) is an ongoing annual population-based survey of prenatal, birth, and postpartum experiences that uses birth certificates to randomly sample mothers with liveborn infants aged 2 to 6 months in 51 participating sites, including 47 states (Shulman et al., 2018). Surveys are conducted via mail with phone follow-up for mail nonrespondents. In addition to core questions asked at every site, each site may select additional questions from a bank of standard questions.

### Analytical Sample

This study used data from New York State and New York City, 2016–2019 (*n* = 7,925). Both New York sites stratify by birthweight in their sampling (Shulman et al., 2018). We excluded respondents who were less than age 18 (*n* = 54), had non-singleton births (*n* = 444), or whose infant was not alive at the time of the survey (*n* = 73). We then excluded respondents who did not have a paid job during pregnancy (*n* = 2,571) or did not plan to return to their pre-pregnancy job (*n* = 850) because they were not surveyed about their leave duration or payment status. We excluded respondents that were less than 12 weeks postpartum at the time of the interview (*n* = 247) to ensure that we could correctly establish a minimum leave duration (< 12 weeks vs. ≥ 12 weeks) for women who were still on maternity leave. Finally, we excluded respondents with incomplete data on postpartum depressive symptoms (*n* = 50) and leave duration and type (paid or unpaid) (*n* = 121) for a final sample size of 3,515. Since the data for this study were deidentified and available for public request from the Centers for Disease Control and Prevention (CDC, 2022), this research was exempt from human subjects review by the University of California, Berkeley Office for Protection of Human Subjects.

### Measures

#### Exposures

Leave characteristics were calculated from PRAMS’ “Occupational Status & Work Place Leave” standard questions. For respondents that had returned to their pre-pregnancy job by the time of the survey, leave duration (weeks) was derived from the question: *How many weeks or months of leave*,* in total*,* did you take?*, with those reporting < 1 week coded as 0. For respondents that had not yet returned to their pre-pregnancy job but intended to return, leave duration was assigned as their weeks postpartum at the time of the survey. Leave duration was examined as both a continuous variable (in weeks) and a binary variable (< 12 weeks vs. ≥12 weeks). We selected 12 weeks as the cut-off based on prior literature (Andres et al., 2016) and because it is a typical maternity leave length in the U.S. (KFF, 2021).

Whether leave was paid was constructed from a question asked of all respondents: *Did you take leave from work after your new baby was born?*, with answer options “I took paid leave from my job”, “I took unpaid leave from my job”, and “I did not take any leave”. In New York City, “I took leave and used Temporary Disability Insurance”, which is a form of paid leave, was a fourth option. Participants checked all options that applied, allowing for creation of a 3-level categorical variable for payment status: fully paid leave (i.e., checked only paid leave or temporary disability), partially paid leave (i.e., checked both paid leave/temporary disability and unpaid leave), no paid leave (i.e., checked only unpaid leave or no leave). We also created a binary variable that combined the fully and partially paid leave categories to create a variable for any paid leave (yes/no).

Finally, we combined leave duration and payment status into a third exposure variable with four levels: paid leave that was ≥ 12 weeks, paid leave that was < 12 weeks, unpaid leave that was ≥ 12 weeks, and unpaid leave that was < 12 weeks.

#### Outcome

Participants completed two questions regarding postpartum depressive symptoms, which were adapted from the Patient Health Questionnaire-2 (PHQ-2): *Since your new baby was born*,* how often have you (1) felt down*,* depressed*,* or hopeless? (2) had little interest or little pleasure in doing things you usually enjoyed?* (Kroenke et al., 2003). Participants responded on a 5-point scale (“always”, “often”, “sometimes”, “rarely”, “never”). We created a binary variable with respondents who checked “always” or “often” for at least one question being characterized as having postpartum depressive symptoms (Bauman, 2020).

#### Covariates

**Fig. 1:**
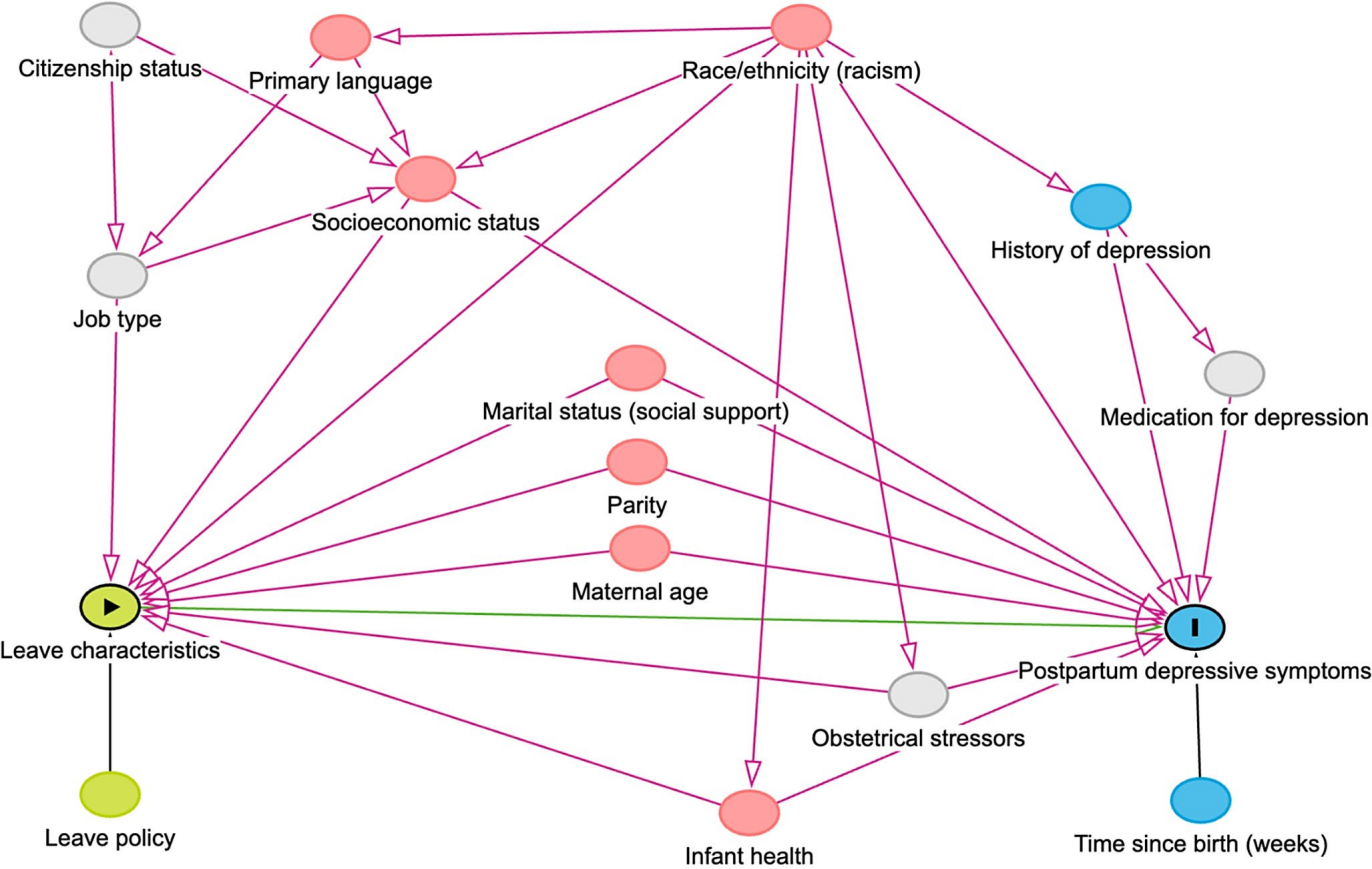
Directed acyclic graph (DAG) of associations between maternity leave characteristics, postpartum depressive symptoms, and measured and unmeasured covariates. Green = exposure, or only associated with exposure; Blue = outcome, or only associated with outcome; Pink = measured confounder; Gray = unmeasured confounder

Covariates were selected a priori based on a directed acyclic graph (Fig. 1) and were categorized as shown in Table 1. Unless otherwise indicated, covariates were used as characterized by PRAMS. Time since birth (weeks rounded down to the nearest integer) was derived from the infant’s reported age at the time of the survey. In New York State, respondents completed the survey in English or Spanish, while New York City also offered Mandarin. Given the low number of responses in Spanish or Mandarin, survey language was operationalized as “English” or “Other”. Household income during the year before the infant was born was collapsed from a 12-level categorical variable into a 5-level variable. Current health insurance was coded as “Private” for those with at least one form of private insurance, “Public” for those with at least one form of public but no private insurance, and “Other” for those with other or no insurance (uninsured was included in “Other” due its small cell size). If respondents experienced depression in the three months before pregnancy and/or during pregnancy, they were categorized as having a history of depression. Based on the year the infant was born, respondents were categorized as having leave before PFL implementation (2016–2017) or after (2018–2019).

**Table 1:** Distribution of selected covariates by leave duration and by any paid leave for PRAMS New York 2016–2019 (*n* = 3,515)^a^

	Total *n* (weighted %)	Leave Duration *n* (weighted %)			Any Paid Leave *n* (weighted %)		
		< 12 weeks	≥ 12 weeks	*p*-value	No	Yes	*p*-value
Time since birth (weeks)^b^	17.1 ± 3.2	17.4 (3.0)	17.0 (3.3)	0.04	17.0 (3.1)	17.2 (3.2)	0.55
Maternal age (years)							
18–24	383 (13.2)	226 (63.3)	157 (36.7)	< 0.001	186 (47.7)	197 (52.3)	< 0.001
25–29	762 (24.2)	310 (46.5)	452 (53.5)		256 (32.1)	506 (67.9)	
30–34	1262 (34.4)	383 (33.9)	879 (66.1)		364 (30.3)	898 (69.7)	
35–39	900 (23.6)	240 (27.4)	660 (72.6)		273 (30.7)	627 (69.3)	
40+	208 (4.5)	62 (34.0)	146 (66.0)		65 (34.5)	143 (65.6)	
Marital status							
Married	2404 (66.5)	753 (34.6)	1651 (65.4)	< 0.001	736 (31.6)	1668 (68.4)	0.02
Other	1111 (33.6)	468 (48.7)	643 (51.3)		408 (36.7)	703 (63.3)	
Maternal race/ethnicity^c^							
Asian/PI	368 (8.5)	118 (33.4)	250 (66.6)	0.002	119 (31.1)	249 (69.0)	0.28
Black	617 (13.4)	175 (31.4)	442 (68.6)		195 (31.8)	422 (68.3)	
White	1845 (58.8)	689 (42.4)	1156 (57.6)		577 (32.7)	1268 (67.3)	
Other	119 (3.1)	45 (41.1)	74 (58.9)		34 (30.9)	85 (69.1)	
Hispanic	560 (16.3)	194 (37.9)	366 (62.1)		216 (38.0)	344 (62.0)	
Survey language							
English	3307 (94.3)	1140 (39.2)	2167 (60.8)	0.77	1040 (32.3)	2267 (67.7)	< 0.001
Other	208 (5.7)	81 (40.6)	127 (59.5)		104 (50.0)	104 (50.0)	
Number of previous live births							
0	1703 (44.2)	520 (35.8)	1183 (64.2)	< 0.001	502 (30.2)	1201 (69.8)	0.001
1	1165 (36.2)	407 (38.9)	758 (61.1)		371 (32.7)	794 (67.4)	
2	373 (11.7)	152 (41.9)	221 (58.1)		156 (42.8)	217 (57.2)	
3+	261 (7.9)	137 (55.6)	124 (44.4)		110 (38.3)	151 (61.6)	
Maternal education (years)							
0–11	155 (5.3)	81 (52.9)	74 (47.1)	< 0.001	85 (51.9)	70 (48.2)	< 0.001
12	509 (16.1)	262 (57.2)	247 (42.8)		213 (42.8)	296 (57.2)	
13–15	854 (24.7)	353 (48.1)	501 (51.9)		322 (37.4)	532 (62.6)	
16+	1988 (54.0)	523 (28.6)	1465 (71.4)		518 (26.7)	1470 (73.3)	
Annual household income ($)							
≤ 20,000	486 (15.5)	240 (56.9)	246 (43.2)	< 0.001	276 (53.8)	210 (46.3)	< 0.001
20,001–40,000	602 (18.4)	272 (50.3)	330 (49.7)		233 (40.8)	369 (59.2)	
40,001–60,000	413 (13.3)	167 (45.3)	246 (54.7)		137 (31.2)	276 (68.8)	
60,001–85,000	344 (10.1)	123 (41.4)	221 (58.6)		104 (30.5)	240 (69.5)	
> 85,001	1520 (42.8)	344 (24.8)	1176 (75.2)		339 (23.8)	1181 (76.2)	
Received WIC during pregnancy							
Yes	1010 (29.8)	475 (53.6)	535 (46.4)	< 0.001	450 (43.7)	560 (56.3)	< 0.001
No	2472 (70.2)	736 (33.2)	1736 (66.8)		682 (28.8)	1790 (71.2)	
Insurance							
Private	2529 (72.5)	755 (33.9)	1774 (66.1)	< 0.001	660 (27.2)	1869 (72.8)	< 0.001
Public	800 (23.4)	395 (56.1)	405 (43.9)		393 (48.1)	407 (51.9)	
Other^d^	152 (4.1)	59 (42.6)	93 (57.5)		81 (57.9)	71 (42.1)	
Preterm birth							
Yes	2845 (94.0)	1018 (39.7)	1827 (60.3)	0.12	911 (33.1)	1934 (67.0)	0.54
No	664 (6.0)	202 (34.6)	462 (65.4)		229 (34.9)	435 (65.1)	
Depression history^e^							
Yes	456 (13.8)	167 (43.5)	289 (56.5)	0.14	169 (34.9)	287 (65.2)	0.53
No	2941 (86.2)	1013 (38.6)	1928 (61.4)		931 (32.8)	2010 (67.2)	
Location							
New York City	2018 (42.6)	616 (33.1)	1402 (66.9)	< 0.001	616 (32.0)	1402 (68.0)	0.21
New York State	1497 (57.4)	605 (43.9)	892 (56.1)		528 (34.3)	969 (65.7)	
Timing of interview^f^							
Before PFL (2016-17)	1876 (55.6)	712 (42.5)	1164 (57.5)	< 0.001	691 (37.5)	1185 (62.6)	< 0.001
After PFL (2018-19)	1639 (44.4)	509 (35.3)	1130 (64.7)		453 (28.2)	1186 (71.9)	
Mean leave duration (weeks)^b^	11.8 ± 0.1	--	--		--	--	
Postpartum depressive symptoms^g^		--	--		--	--	
Yes	408 (10.6)	--	--		--	--	
No	3107 (89.4)	--	--		--	--	
Leave duration		--	--		--	--	
< 12 weeks	1221 (39.3)	--	--		--	--	
≥ 12 weeks	2294 (60.7)	--	--		--	--	
Payment status		--	--		--	--	
Unpaid	1144 (33.3)	--	--		--	--	
Partially paid	508 (14.1)	--	--		--	--	
Fully paid	1862 (52.6)	--	--		--	--	

^a^ Sample sizes are smaller for some covariates due to missing data, and all Ns are unweighted

^b^ Mean ± Standard Deviation

^c^ Asian/PI, Black, and White are non-Hispanic; Other includes non-Hispanic Other and those whose ethnicity (Hispanic vs. non-Hispanic) was unknown

^d^ Other includes those who were uninsured

^e^ Categorized as “Yes” if experienced depression in 3 months before pregnancy and/or during pregnancy

^f^ Paid Family Leave (PFL) legislation was first implemented on January 1, 2018

^g^ “Yes” includes those who answered “Always” or “Often” to at least 1 of the 2 depression screening questions

### Statistical Analysis

All analyses were performed using survey weights to account for the complex sampling design, including, for example, the potential for this sample to have a higher risk of PPD due to the oversampling of low-birth-weight infants (Shulman et al., 2018; Vigod et al., 2010). Because of the PRAMS complex weighting scheme, the calculated percentages represent population estimates for women who gave birth in the state of New York. Characteristics of leave duration, payment status of leave, postpartum depressive symptoms, and covariates were described, and chi-squared tests and independent sample t-tests were conducted to examine the associations between leave exposures and covariates. Logistic regression was used to examine the crude and adjusted associations between the leave exposures and postpartum depressive symptoms. In the adjusted models, we controlled for all covariates identified a priori. We conducted four logistic regression models using the following exposure variables: (1) leave duration (continuous) and any paid leave (yes/no), (2) leave duration (< 12 vs. ≥12 weeks) and any paid leave (yes/no), (3) a combined leave duration and payment status variable, and (4) payment status (paid only, paid and unpaid, unpaid only). Given the potential for a non-linear relationship between leave duration and depressive symptoms, we performed a sensitivity analysis analyzing leave duration as a continuous variable separately for those with leave < 12 weeks and leave ≥ 12 weeks. We also tested for interaction between paid leave and duration of leave (as both a continuous and binary variable) using cross-product terms; however, because we did not observe any statistically significant interaction, models without interaction terms are presented. Analyses were performed using Stata (17.0) and R (4.1.1) with the package ‘survey’ for applying the weights.

## Results

Table 1 shows the characteristics of the study sample using weighted percentages and unweighted number of observations (*N* = 3,515). Overall, an estimated 60.7% of New York’s birthing population had a leave duration of ≥ 12 weeks, 66.7% had at least some paid leave, and 10.6% experienced postpartum depressive symptoms. Over half (58.8%) of respondents identified as non-Hispanic White, and 42.8% reported an annual household income above $85,001. On average, respondents were 17.1 ± 3.2 weeks postpartum at the time of the survey.

Characteristics associated with leave duration and any paid leave are also shown in Table 1. Leave ≥ 12 weeks and paid leave were more common in respondents who were older, married, and of higher socioeconomic status (i.e., higher education, higher income, private insurance, and not being eligible for Women, Infants, & Children (WIC) Supplemental Nutrition Program). Non-Hispanic Black respondents were most likely to have leave ≥ 12 weeks (68.6%) followed by non-Hispanic Asian/Pacific Islander respondents (66.6%), but there were no statistically significant differences for payment status by race/ethnicity. As expected, leave ≥ 12 weeks and paid leave were more common during the 2 years after the implementation of New York’s PFL policy than in the 2 years before.

In logistic regression analyses (Table 2), receiving no paid leave was significantly associated with an increased odds of postpartum depressive symptoms in both crude and adjusted models (model 1: aOR = 1.41 [95% CI: 1.04–1.93]; model 2: aOR = 1.42 [95% CI: 1.04–1.95]). However, duration of leave (as a continuous variable [model 1] or dichotomous variable [model 2]) was not associated with depressive symptoms.

**Table 2:** Crude and adjusted associations of leave duration and payment status with postpartum depressive symptoms, PRAMS New York 2016–2019

	Postpartum Depressive Symptoms	
	Crude OR (95% CI)^a^	aOR (95% CI)^b, c^
*Model 1*		
Leave duration (weeks)	0.99 (0.96, 1.01)	0.99 (0.97, 1.02)
Any paid leave		
Yes	Ref	Ref
No	1.42 (1.07, 1.88)*	1.41 (1.04, 1.93)*
*Model 2*		
Leave duration		
≥ 12 weeks	Ref	Ref
< 12 weeks	1.13 (0.85, 1.51)	1.05 (0.76, 1.45)
Any paid leave		
Yes	Ref	Ref
No	1.42 (1.07, 1.89)*	1.42 (1.04, 1.95)*
*Model 3*		
Payment status and leave duration combined		
Paid ≥ 12 weeks	Ref	Ref
Paid < 12 weeks	1.35 (0.94, 1.94)	1.22 (0.82, 1.82)
Unpaid ≥ 12 weeks	1.72 (1.22, 2.43)*	1.68 (1.16, 2.42)*
Unpaid < 12 weeks	1.52 (1.03, 2.23)*	1.42 (0.89, 2.26)

OR, odds ratio; aOR, adjusted odds ratio

^a^*n* = 3515

^b^*n* = 3172

^c^ Adjusted for time since birth [weeks], maternal age, marital status, race/ethnicity, survey language, number of previous births, education, household income, participation in WIC during pregnancy, insurance, preterm birth, depression history, location, and timing of interview

* *p* < 0.05

When we combined leave duration and payment status into a single variable (model 3), we similarly observed increased odds of depressive symptoms in both groups who received no paid leave compared to women who received paid leave for ≥ 12 weeks in crude models. After adjusting for selected covariates, only the increased odds among the group with unpaid leave for ≥ 12 weeks remained statistically significant (aOR = 1.68, 95% CI: 1.16–2.42) compared to the reference group.

When we further examined payment status as unpaid, partially paid, or fully paid (Table 3), there was no significant association for depressive symptoms between the group that had partially paid leave compared to the group that was fully paid. However, having unpaid leave was associated with an increased odds of depressive symptoms compared to having fully paid leave (aOR = 1.49, 95% CI: 1.07–2.06).

**Table 3:** Crude and adjusted associations between payment status of leave (3-level variable) and postpartum depressive symptoms, PRAMS New York 2016–2019

	Postpartum Depressive Symptoms	
	Crude OR (95% CI)^a^	aOR (95% CI)^b, c^
*Model 4*		
Payment status		
Fully paid leave	Ref	Ref
Partially paid leave	0.99 (0.66, 1.49)	1.17 (0.74, 1.85)
Unpaid leave	1.43 (1.06, 1.92)*	1.49 (1.07, 2.06)*

^a^*n* = 3514

^b^*n* = 3171

^c^ Adjusted for leave duration (< 12 weeks vs. ≥ 12 weeks weeks) and for all covariates (time since birth [weeks], maternal age, marital status, race/ethnicity, survey language, number of previous births, education, household income, participation in WIC during pregnancy, insurance, preterm birth, depression history, location, and timing of interview)

* *p* < 0.05

In sensitivity analyses examining < 12 weeks and ≥ 12 weeks of leave separately (Supplemental Table S1), we still saw no significant associations between leave duration as a continuous variable and depressive symptoms.

## Discussion

In a representative sample of postpartum women in New York from 2016 to 2019, we found that not receiving any paid maternity leave was associated with a 40% increase in the odds of experiencing postpartum depressive symptoms compared to having paid leave. Shorter maternity leave duration (independent of payment status) was not associated with postpartum depressive symptoms. Receiving even some paid leave appeared to be protective: the odds of depressive symptoms were not significantly higher among women with partially paid leave compared to those with fully paid leave. Increased odds were only seen among those who received no paid leave at all, including among those with longer unpaid leave.

In contrast to existing literature (Andres et al., 2016), we did not find a significant association between shorter leave duration and PPD. One previous study found that the association between leave duration and PPD was significant among those who had returned to work by 12 weeks but not among those who returned to work after 12 weeks, but this was not seen in our data (Kornfeind & Sipsma, 2018). Another study found that the relationship between leave duration and depressive symptoms was U-shaped, such that longer leave duration was associated with lower depressive symptoms until 6 months, at which point it was associated with increased depression (Dagher et al., 2014). However, there were few people in our sample who had taken 6 or more months of leave (*n* = 71) at the time of interview, as PRAMS only samples women up to 6 months postpartum. Our lack of association with leave duration may reflect the fact that we had more recent data than prior studies or that we controlled for variables likely to be strong confounders but had not been fully accounted for in some prior studies (Petts, 2018). Importantly, our study controlled for whether the leave was paid in addition to the duration of leave. However, it is also possible that our lack of association was related to measurement error in the leave duration variable and the cut-off point of 12 weeks that we selected.

Nevertheless, our finding that having access to paid leave is protective against postpartum depressive symptoms regardless of leave duration aligns with prior literature (Aitken et al., 2015; Van Niel et al., 2020). Paid leave theoretically mitigates economic stress, an established mechanism underlying PPD (Payne & Maguire, 2019). Our study suggests that being paid during leave may be more important for maternal mental health than overall duration of leave. In fact, we found the highest odds of postpartum depressive symptoms among women with unpaid leave of longer duration. Those with longer unpaid leave may experience greater levels of economic stress or greater concerns about job security than those with unpaid leave who return to work earlier. Additionally, our findings regarding leave duration may be related to the ‘enhancement hypothesis’, which posits that having multiple roles, such as being a working parent, can benefit individuals through pathways such as increased self-esteem and social support that people may derive from working (Hyde & McKinley, 1993). It is possible returning to work earlier provides some individuals with these protective factors.

This study has a number of limitations. First, it is possible that there was measurement error in our exposure variables. We suspect that some respondents mistakenly reported their leave duration in months instead of weeks. However, less than 1% of continuous duration responses would have been impacted by this and even fewer responses would have been misclassified in our binary duration exposure. Additionally, among those who reported having partially paid leave, we do not know how much time was paid. As both the exposure and outcome were self-reported, respondents may have had challenges or biases with recall. The PHQ-2 is a widely used depression screener that has shown good sensitivity and specificity for PPD (Bennett et al., 2008); however, it only contains two questions and may not accurately characterize depression symptoms. Additionally, it is possible that people with more severe or recent depressive symptoms are less likely to participate in the PRAMS surveys and were therefore left out of our sample. We also may be missing cases for those who participated before developing depressive symptoms; however, the prevalence of postpartum depressive symptoms in our study aligns with prior literature (Anokye et al., 2018; O’Hara & McCabe, 2013). Another limitation of this study is that we likely had unmeasured confounding, including information about respondents’ job characteristics (Schwab-Reese et al., 2017) and other measures of social support, such as whether or not the mother’s partner took leave. Unlike some prior studies (Kornfeind & Sipsma, 2018), we did not have a measure of respondents’ satisfaction with their maternity leave experience, which could have helped contextualize our findings.

This study also has several strengths. Compared to previous research, our study uses more recent data that is likely to be more representative of the current workforce, at least in New York. Our study sample is representative of the population of New York, a racially diverse state that had recently implemented a policy of 12-weeks of paid family leave at the time of the study. Although the New York population may not be generalizable to other states, the racial and ethnic composition of our sample is similar to the United States as a whole (“U.S. Census Bureau QuickFacts,” n.d.), with more than 40% of our sample being people of color. Our study has a larger sample size than many previous studies, which may have increased our power. To our knowledge, our study is one of the few investigations of postpartum mental health to examine the exposures of leave duration and paid leave both individually and in combination within the U.S. context. Unlike previous studies (Jou et al., 2018; Mandal, 2018), our analyses distinguished between fully and partially paid leave, which may have relevant implications for policy.

Our findings have important implications for clinical practice and parental leave policy. Providers and policymakers should be mindful that birthing parents without access to paid leave may be more likely to experience PPD, as demonstrated by our study and prior studies (e.g., (Aitken et al., 2015). Although this study did not find a significant association between leave duration and postpartum depressive symptoms, longer leave duration may relate to other outcomes outside the scope of this study (Andres et al., 2016). In alignment with previous studies (e.g., Van Niel et al., 2020), this study suggests that having access to paid leave may reduce risk of postpartum depression, which can have long-term health effects for both maternal and child outcomes (Slomian et al., 2019). Future studies can help to identify which communities could benefit most from paid leave and inform paid leave policies.

## Electronic supplementary material

Below is the link to the electronic supplementary material.

Supplementary Material 1

## Data Availability

Available for public request from the Centers for Disease Control and Prevention.

## Code Availability

Code in Stata and R available upon request from corresponding author.
