# Supplementary material for: Association of Maternity Leave Characteristics and Postpartum Depressive Symptoms among Women in New York
Source: Matern Child Health J. 2024 Oct 5;28(11):1990–9. doi: 10.1007/s10995-024-03997-1 (PMC11534827; doi:10.1007/s10995-024-03997-1)
Supplement: Supplementary file 1 — Supplementary Material 1 [file 10995_2024_3997_MOESM1_ESM.docx]

| Supplemental Table S1. Sensitivity analysis: crude and adjusted associations between leave duration and postpartum depressive symptoms, PRAMS New York 2016-2019. | | | | |
| --- | --- | --- | --- | --- |
|  |  | **Postpartum Depressive Symptoms** | |  |
|  |  | Crude OR (95% CI) | aOR (95% CI)^a^ |  |
| *Respondents with <12 weeks of leave (N=1221)* | | |  |  |
| Leave duration (weeks) | | 0.95 (0.87, 1.03) | 0.95 (0.86, 1.05) |  |
|  |  |  |  |  |
| *Respondents with ≥12 weeks of leave (N=2294)* | | |  |  |
| Leave duration (weeks) | | 0.99 (0.96, 1.02) | 0.98 (0.94, 1.02) |  |

^a^ Adjusted for any paid leave (yes/no) and all covariates (time since birth [weeks], maternal age, marital status, race/ethnicity, survey language, number of previous births, education, household income, participation in WIC during pregnancy, insurance, preterm birth, depression history, location, and timing of interview).
